# Inflow Occlusion Combined With Bleomycin Sclerotherapy for Management of Macro/Mixed Cystic Lymphatic Malformation in Children

**DOI:** 10.3389/fped.2021.749983

**Published:** 2021-09-22

**Authors:** Tao Han, Yi Ji, Jie Cui, Weimin Shen

**Affiliations:** Department of Burns and Plastic Surgery, Children's Hospital of Nanjing Medical University, Nanjing, China

**Keywords:** cystic lymphatic malformation, inflow occlusion, bleomycin, indocyanine green, lymphography

## Abstract

**Background:** The link between cystic lymphatic malformation (cLM) and normal lymphatic system has become the focus of research. This study aimed to assess the outcomes of indocyanine green (ICG) lymphography-guided inflow occlusion combined with bleomycin sclerotherapy for the management of macro or mixed cLM in children.

**Methods:** Between June 2018 and October 2020, inflow occlusion combined with bleomycin sclerotherapy was performed in 81 cLM patients (age range from 6 months to 8 years). All cases were evaluated by the following parameters: cLM location, histological typing, number of afferent lymph vessels, dermal backflow, curative effects, treatment frequency, and postoperative complications. The duration of postoperative follow-up was from 10 to 16 months.

**Results:** All cLM cases could be found with at least one lymphatic inflow. Excellent outcomes were observed in 68 cases (84.0%), 11 cases (13.6%) experienced good outcomes, and two (2.5%) cases had fair outcome. No case experienced repeated treatment for more than three times. Wound infection, fever, and scar hyperplasia were the independent adverse events, which were managed by symptomatic treatment.

**Conclusion:** Inflow occlusion combined with bleomycin sclerotherapy renders a safe and efficient approach for the management of macro or mixed cLM.

## Introduction

Cystic lymphatic malformations (cLM) are nonmalignant vascular malformations of the lymphatic system, characterized by the dilated cysts lined by lymphatic endothelial cells (1, 2). According to the classification system of the International Society for the Study of Vascular Anomalies (ISSVA), cLM can be histologically categorized into three subtypes, including macrocystic, microcystic, and mixed cystic. It may change in size resulting from local infection, intracapsular hemorrhage or trauma, and even resolve spontaneously in rare cases (3–5). cLM has been managed by multidisciplinary teams worldwide, each according to their own preferences regarding therapeutic intervention approaches, including surgical resection, sclerotherapy, oral sirolimus, radiofrequency ablation, and laser therapy (6–11). In recent years, sclerotherapy plays a main role in the management of macro cLM in terms of safety and aesthetics. However, given that some of cLMs with inflow pattern are connected to lymphatic system, posttreatment accumulation of lymph in the lesion could always be observed, which may eventually lead to local recurrence and repeated sclerotherapy (12, 13). For improved outcomes, the link between cLM and normal lymphatic system has gradually become the focus of research nowadays. Recently, indocyanine green (ICG) fluorescence imaging system has gained increasing interest with its significant detection of both inflow and outflow of cLM and is considered to be useful for the interventions of cLM (14).

With our previous ICG lymphography study, we found that only one afferent lymph vessel was detected in most cases of macro cLM and mixed cLM, while all micro cLM cases had more than two inflows. Based on our experience and relevant literatures, we herein hold the thought that it is feasible for macro/mixed cLM cases to perform inflow occlusion for blocking the link to the lymphatic system, which could be beneficial for reducing lymph accumulation postoperatively. The purpose of this study was to present our experience in performing inflow occlusion combined with bleomycin sclerotherapy as an alternative management of macro/mixed cLM in children.

## Patients and Methods

### Patients

Between June 2018 and October 2020, we conducted a prospective research for this treatment in 81 children diagnosed with macro or mixed cystic LM in our center. The male-to-female ratio was 48:33, with an age range of from 6 months to 8 years. Preoperative physical examination and MRI were performed to determine both region and extent of cLM lesions. All patients were evaluated by the following parameters: cLM location, histological typing, number of afferent lymph vessels, dermal backflow, curative effects, treatment frequency, and postoperative complications. Adverse events included wound infection, fever, scar hyperplasia, and temporary ICG-related pigmentation. The inclusion criteria included the following: (1) no previous intervention (including surgery or sclerotherapy), (2) rapidly involving symptoms, (3) macro/mixed cLM confirmed by preoperative MRI and postoperative pathology, and (4) inflows from lymphatic system confirmed by ICG lymphography. The exclusion criteria included the following: (1) history of iodine allergy, (2) micro cLM, (3) syndromic cLM (e.g., Klippel–Trenaunay syndrome, Gorham–Stout disease), and (4) isolated cLM (no inflow detected). All study protocols were conducted subsequent to receiving consents from the parents of cLM patients. This clinical study was approved by the Ethics Committee of the Children's Hospital of Nanjing Medical University.

### Surgical Technique

Bleomycin powder (15 mg, Hisun Pfizer Co., Ltd., Hangzhou, China) was reconstituted with 15 ml of normal saline, yielding a 1-mg/ml concentration. The amount of bleomycin injection depended on the size of cLM. The dose of injection was 0.5–1 mg/kg, and the maximum dose was limited to 15 mg per session.

**Figure 1:**
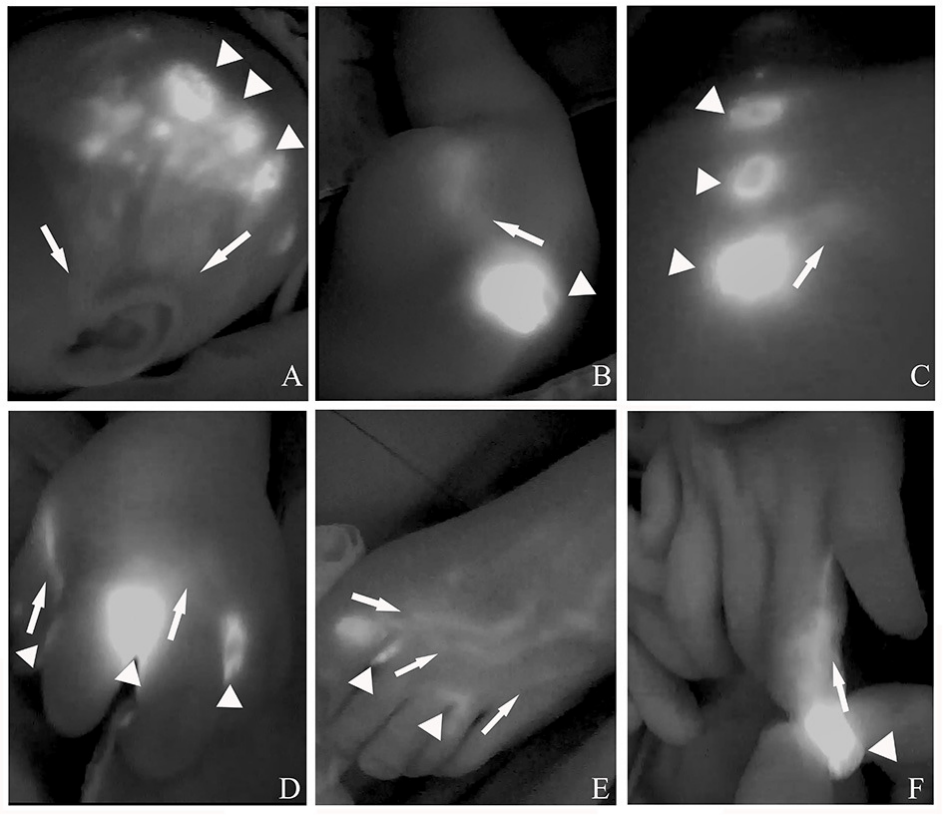
The selection of indocyanine green (ICG) injection points for detecting inflows of cystic lymphatic malformations (cLM) at the **(A)** cervicofacial region, **(B)** chest, **(C)** abdomen or back, **(D)** upper extremities, **(E)** lower extremities, and **(F)** digits. *Arrow head*, lymph vessel; *triangle*, ICG injection point.

Under general anesthesia, both intradermal and subcutaneous injections of ICG were performed in multipoints distal to the cLM lesion. At each injection site, 0.05 to 0.1 ml of ICG solution (concentration: 2.5 mg/ml, Dandong Yichuang, China) was administered. The maximum dose of ICG was 0.5 mg/kg per session. To minimize the possibility that injected ICG does not enter the lesion and stays in the soft tissue around the injection site or in the lymph node, we routinely performed a 10-min local massage from the distal to proximal regions. ICG lymphography was performed by using near-infrared fluorescence imaging system (Mingde Medical Diagnosis Inc., Langfang, China) with its optical handheld probe. The probe, perpendicular to the skin, was covered with a sterile drape for adjusting its position at a distance of 10 cm. The tissue penetration was limited to 10 mm for visualization, and real-time video images captured during the procedure were stored for later review. The injection points for detecting inflows of cLM at specific locations were summarized as follows: (1) cLM at the cervicofacial region: temporal scalp, (2) cLM at the chest: ipsilateral para-areolar area, (3) cLM at the abdomen or back: midline area, 3–5 cm distal to the cLM, (4) cLM at the upper extremities: first and third web spaces, ulnar and radial volar wrist area, (5) cLM at the lower extremities: first and third web spaces, and (6) cLM at digits: both sides of the tiptoe/fingertip, nail bed (Figure 1).

**Figure 2:**
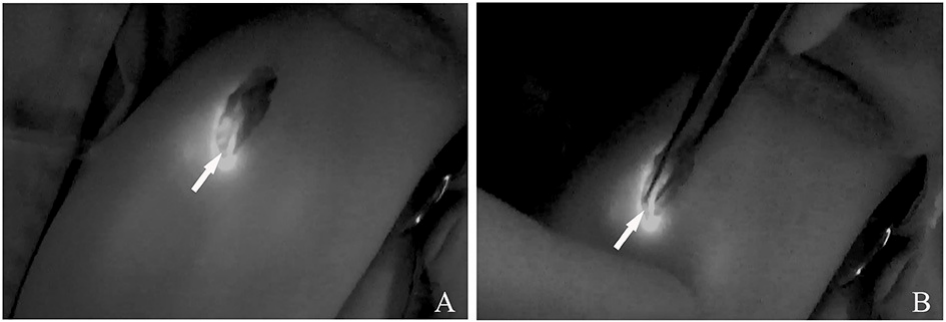
**(A)** ICG lymphography indicates the localization of the afferent lymph vessels. **(B)** Inflow occlusion is conducted by bipolar electrocoagulation. *Arrow head*, lymph vessel.

With completion of ICG injections and observations, we confirmed both location and number of inflows. The detected inflows were visualized gradually from distal to proximal region, and a skin incision was performed in the region where afferent lymph vessels drained into the cLM lesion. The lymph vessels were separated from the incision and confirmed by ICG lymphography (Figure 2A). Subsequently, identified inflows were occluded by bipolar electrocoagulation with the power of 50 W (Figure 2B). A pair of scissors was used to pass through the cystic wall, and lymph in the lesion was drained by suction, and then a small part of the cystic tissue was resected for postoperative pathology. For reducing the postoperative accumulation of lymph, fluorescence imaging probe (without another ICG injection) was used again to rule out both inflow visualization and lymphatic leak with the focus on the previous detected site.

Thereafter, we placed a drainage tube into the cyst from the incision, and bleomycin was then administered through this tube. After 2 h, a syringe of negative pressure was then connected to the intralesional drainage tube. It was removed on the fifth to seventh day of postoperation when no lymph could be aspirated. For all patients enrolled in our study, the duration of postoperative follow-up was from 10 to 16 months (mean length: 12.4 months).

### Curative Effect Evaluation

Both physical examination and MRI images were compared preoperatively and postoperatively; the effective response was evaluated by assessing the reduction rate of the lesion (reduced size/pre-op size × 100%) as follows: (1) excellent, defined as a reduction rate of more than 90%, (2) good, defined as a reduction rate from 75 to 90%, (3) fair, defined as a reduction rate from 50 to 75%, and (4) poor, defined as a reduction rate of <50% (15).

## Results

**Figure 3:**
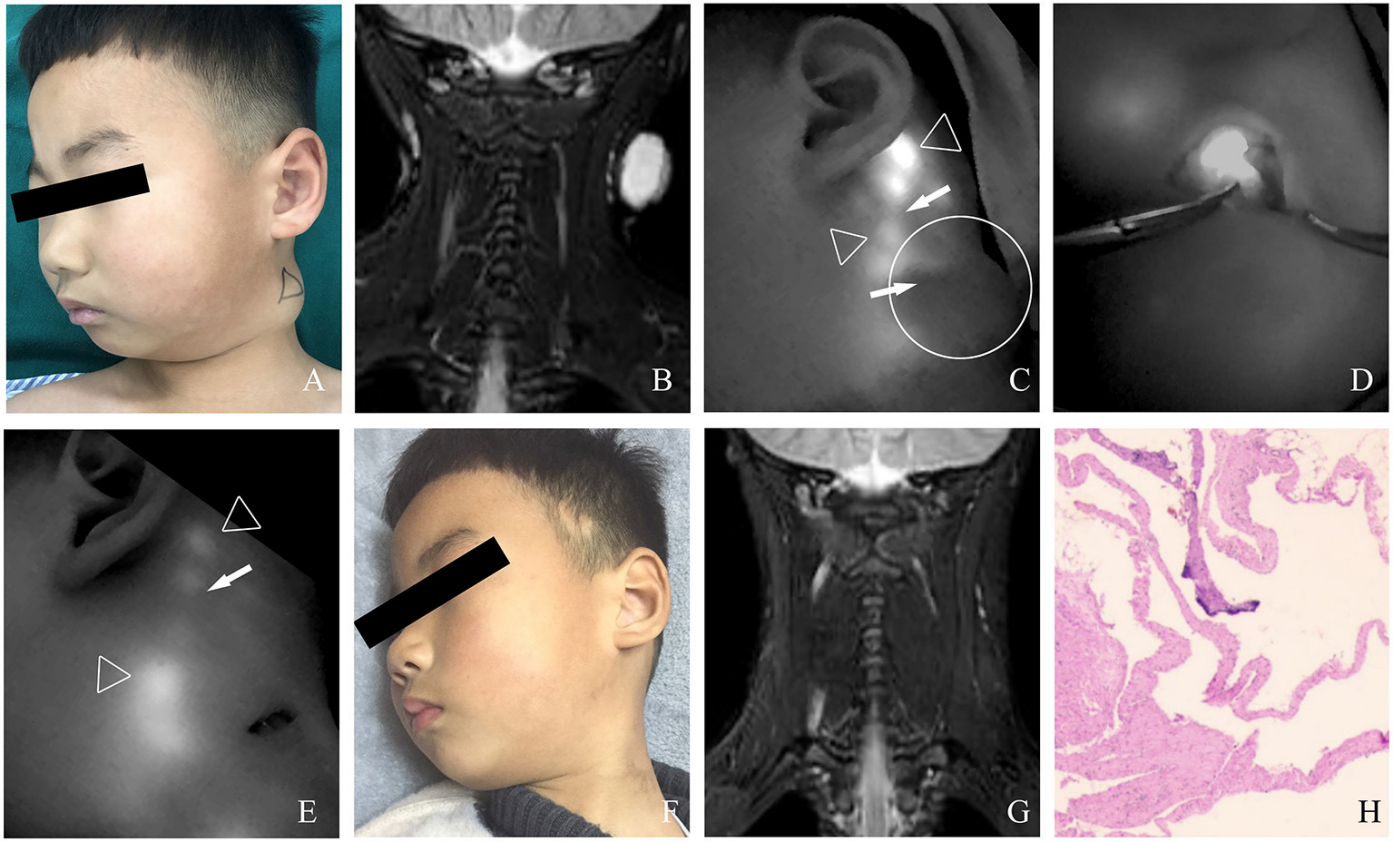
**(A)** A 4-year-old male patient with macro cLM of left neck preoperatively. **(B)** MRI preoperatively. **(C)** A normal lymph vessel from the left temporal scalp ran into the posterior auricular region, from which another afferent lymph vessel was observed flowing into the lesion cite. **(D)** The cLM lesion confirmed by ICG lymphography during surgery. **(E)** No inflow visualization or lymphatic leak on the previous detected site after inflow occlusion. **(F)** Ten months postoperatively, complete regression of cLM met the criterion of excellent curative effect. **(G)** MRI postoperatively. **(H)** Pathology postoperatively. *Arrow head*, lymph vessel; *circle*, region of cLM; *hollow triangle*, lymph node.

**Figure 4:**
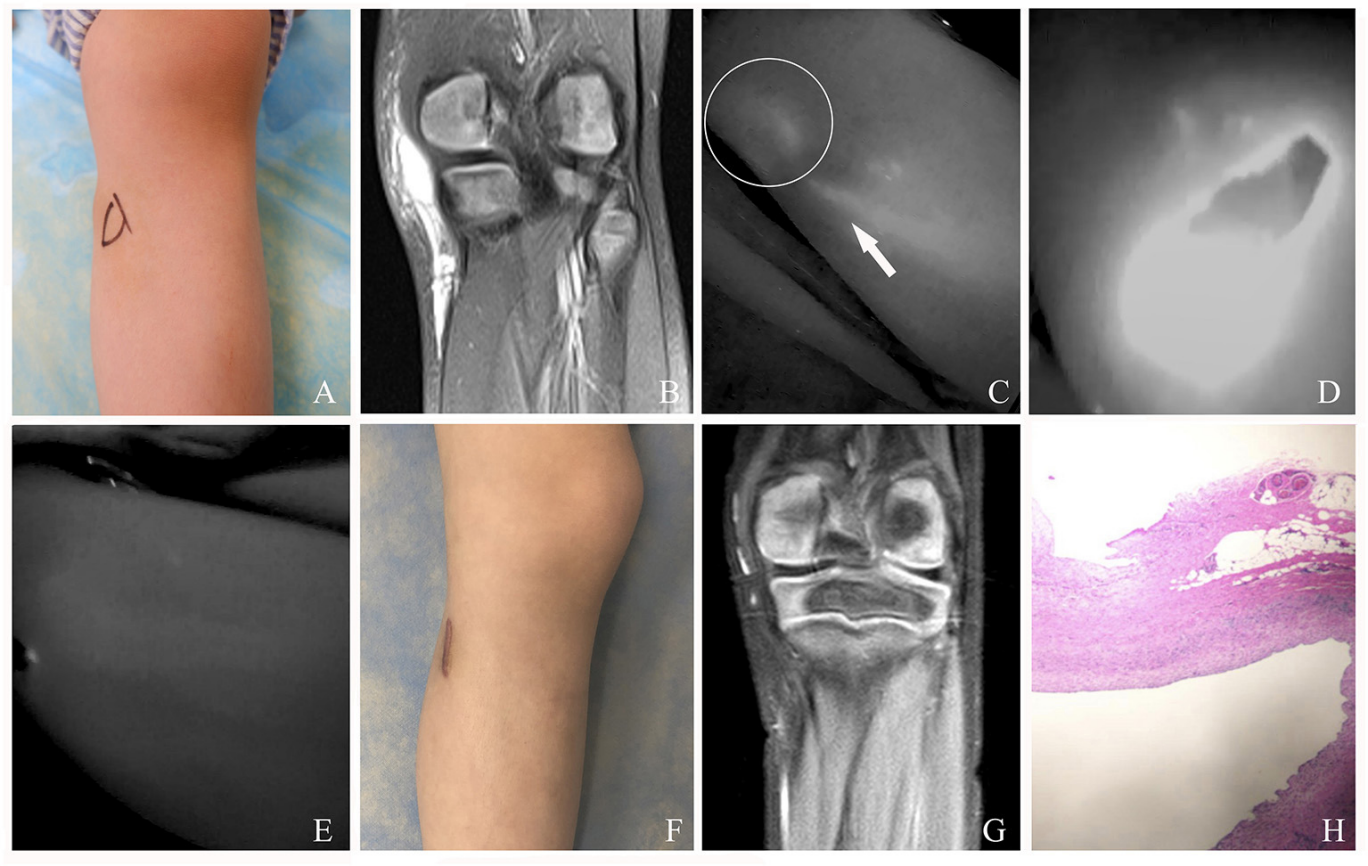
**(A)** A 5-year-old female patient with mixed cLM of left knee preoperatively. **(B)** MRI preoperatively. **(C)** ICG finding with one afferent lymph vessel draining into a lesion. **(D)** The cLM lesion confirmed by ICG lymphography during surgery. **(E)** No inflow visualization or lymphatic leak on the previous detected site after inflow occlusion. **(F)** Twelve months postoperatively, complete regression of cLM met the criterion of excellent curative effect. Remarkably, this patient suffered from skin scar hyperplasia. **(G)** MRI postoperatively. **(H)** Pathology postoperatively. *Arrow head*, lymph vessel; *circle*, region of cLM.

**Figure 5:**
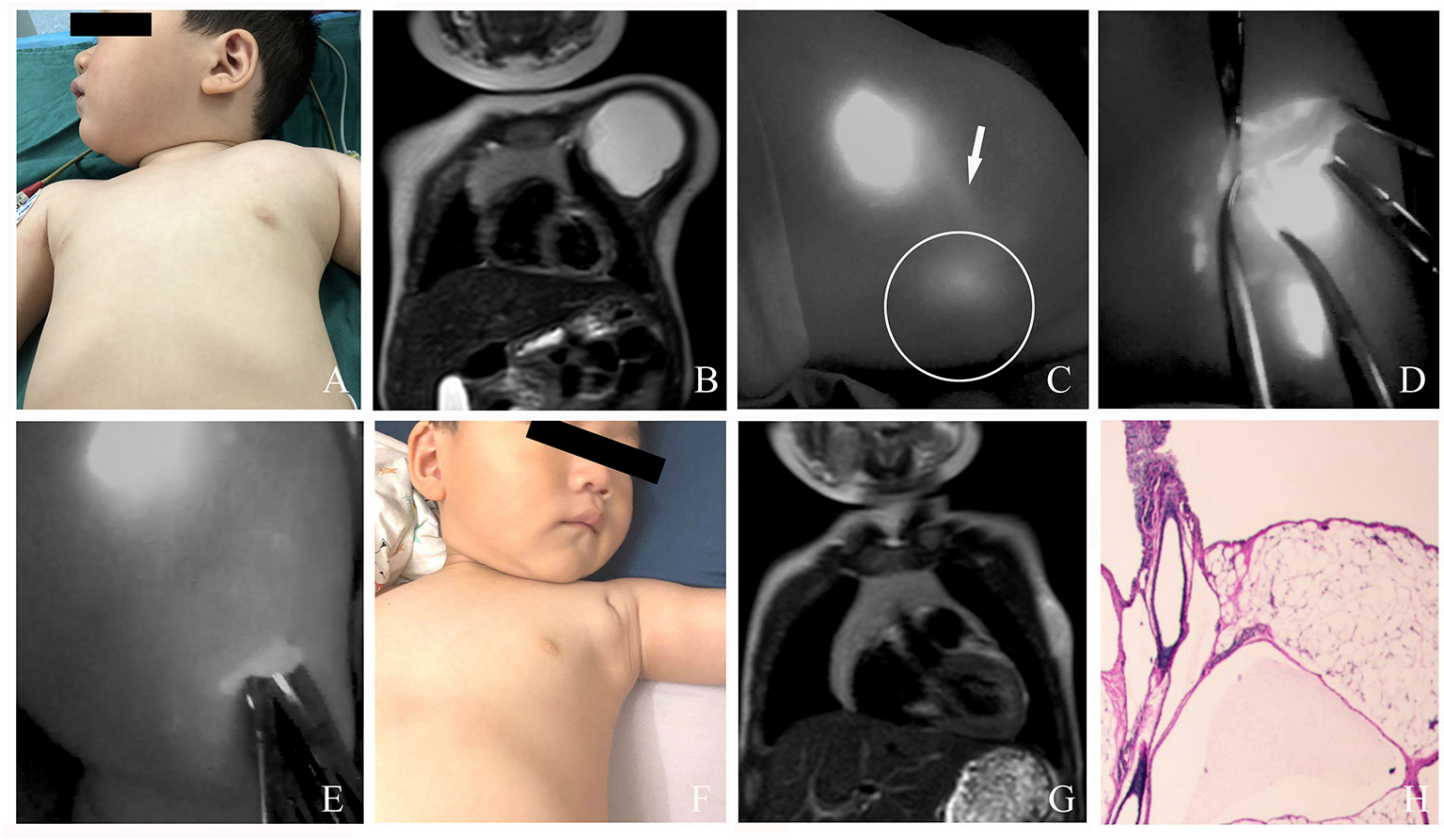
**(A)** A 2-year-old male patient with macro cLM of the left chest preoperatively. **(B)** MRI preoperatively. **(C)** ICG finding with one afferent lymph vessel draining into a lesion. **(D)** The cLM lesion confirmed by ICG lymphography during surgery. **(E)** No inflow visualization or lymphatic leak on the previous detected site after inflow occlusion. **(F)** Ten months postoperatively, complete regression of cLM met the criterion of excellent curative effect. **(G)** MRI postoperatively. **(H)** Pathology postoperatively. *Arrow head*, lymph vessel; *circle*, region of cLM.

The characteristics and outcomes of all 81 cases with macro or mixed cLM are summarized in Table 1. After injection of ICG, the afferent lymph vessels were gradually visualized from the distal to proximal region, and eventually drained into cLM lesions. As shown by ICG lymphography, no morphological or structural abnormality in afferent lymph vessels was detected. Remarkably, all cLM cases could be found with at least one lymphatic inflow, but without outflow. After occlusion, the afferent lymph vessels could not be visualized on the previous detected site. Neither lymphatic leak nor regeneration of the collateral pathway was observed postoperatively. One mixed cLM case with lymphatic dermal backflow was revealed in this study. This might be caused by the high pressure or dysfunction of lymph vessels, and ICG/lymph flowed back abnormally to the dermal tissue space. Sixty-eight cases (84.0%) with excellent clinical outcomes resulted in notably improved appearance (Figures 3–5), 11 cases (13.6%) experienced partial reduction in the size of cLM lesion, two cases (2.5%, one at the right scapular region, one at the right upper extremity) with fair outcomes required subsequent treatment, and their follow-up was still in progress. Besides, majority of our cases (91.4%) experienced only one session of this treatment. Repeated treatments were needed in 8.6% of cases, in which inflow was no longer visualized by ICG lymphography. Therefore, inflow occlusion was not necessary to perform again, and only bleomycin sclerotherapy was repeated in a subsequent procedure. It is worth noting that no case in our research was treated for more than three times (Table 2).

**Table 1:** Characteristics and outcomes of patients.

**Demographics**	**Patients (*n* = 81)**	**%**
Gender		
Male	48	59.3
Female	33	40.7
Age group (years)		
<1	27	33.3
1~3	40	49.4
>3	14	17.3
Location		
Cervicofacial region	41	50.6
Trunk	29	35.8
Extremities	11	13.6
Histological typing		
Macro cLM	50	61.7
Mixed cLM	31	38.3
Inflow (no.)		
1	74	91.4
2	7	8.6
Dermal backflow	1	1.2

*cLM, cystic lymphatic malformation*.

**Table 2:** Outcomes of the postoperative period.

**Results**	**Patients (*n* = 81)**	**%**
Curative effects		
Excellent	68	84.0
Good	11	13.6
Fair	2	2.5
Poor	0	0
Treatment frequency (times)		
1	74	91.4
2–3	7	8.6
>3	0	0

In this study, fever (8.3%) and scar hyperplasia (8.3%) were the independent adverse events, which were managed by symptomatic treatment. All patients experienced temporary ICG-related pigmentation in the injection sites, which faded away gradually within 1 month postoperatively. There was no ICG allergy or secondary lymphedema occurring in all cases (Table 3).

**Table 3:** Postoperative complications.

**Subject**	**Patients (*n* = 81)**	**%**
Fever	2	2.5
Scar hyperplasia	2	2.5
Temporary ICG-related pigmentation	81	100

*ICG, indocyanine green*.

## Discussion

Given a localized lesion, surgery resection and sclerotherapy are always the main choices for the management of both macro and mixed cLM (3, 16–19). However, a series of cLM cases treated by surgery or sclerotherapy suffer from limited efficacy or local recurrence, especially for the cLM at the cervicofacial region, axilla, and joints. The uncertain border of cLM at these regions makes radical resection very difficult, and the lymph from afferent vessels accumulates again after sclerotherapy, leading to multiple sessions and even focal relapse of cLM (20, 21). Therefore, exploring the link between cLM and the lymphatic system, could gain insight into its pathogenesis and, furthermore, offers a new thought for targeted therapeutic approaches.

MRI examination, as the principal method of diagnosing cLM, is widely employed in evaluation of the lesion and its relationship with adjacent structures (22, 23). MRI is particularly useful for indicating its border, septum, lesion range, and content within the cysts, with low-signal intensity on T1 and high-signal intensity on T2. However, the peripheral lymph flow around cLM cannot be demonstrated by MRI, which hinders the understanding of changes in the local lymphatic drainage in cLM patients. Therefore, it is necessary to combine lymphography imaging to improve the assessment of cLM.

ICG, as a safe and stable fluorescent dye, can be easily absorbed by lymphatic capillaries (24). The original applications of ICG lymphography in the field of lymphatic surgeries were to detect lymph flow patterns or to confirm the treatment outcomes in both primary and secondary lymphedema (25–27). Recently, its capability to penetrate deep into soft tissue (up to 10 mm for visualization) opens the door to real-time intraoperative navigation for the management of cLM. Kato et al. (13, 14, 28) reported that the flow patterns of cLM can be classified into four types, and flow-oriented venous anastomosis for creating drainage bypass was considered an effective surgical approach for micro or mixed cLM. Kubota et al. (29) reported an adult case with axillary cLM that was successfully treated with ICG lymphography for visualization of lymph vessel and lesion. Additionally, Shirota et al. (30) revealed that following ICG injection into the skin above the lesion, the precise surgical margins of cLM could be identified, which, in turn, aid in complete resection.

In the study presented here, we performed intraoperative ICG lymphography to detect afferent lymph vessels. The skin incision was selected in the region where afferent lymph vessels drained into the lesion, so that it was beneficial for exposure of both inflow and cLM. For stable postoperative outcome, we performed inflow occlusion combined with bleomycin sclerotherapy, which had not been previously reported in the treatment of macro or mixed cLM. The principle behind this technique is to block the communication between the lesion and lymphatic system, and to induce shrinkage of the cysts. Fluorescence imaging probe was intraoperatively used to assess inflow occlusion and ruled out lymphatic leak, so as to reduce the postoperative accumulation of lymph in the lesion.

It is worth mentioning that, it is not feasible to ligate the inflow without lesion resection, which may lead to a regeneration of the collateral pathway postoperatively (13). Besides, for patients with micro cLM, our technique is not the ideal treatment modality of choice. Due to multiple small inflows observed in most micro cLM cases, occluding all of them may impede normal lymphatic drainage and cause secondary lymphedema subsequently.

The selection of management of cLM relies on lesion location, patient preference, and experience of the surgeon. Sclerotherapy has emerged as a first-line treatment for macro cLM in the cervicofacial region with its advantages, including simple operation, less trauma, and fewer side effects (31, 32). Bleomycin, an antibiotic derivative with cytostatic properties, induces obliteration and fibrosis of cysts by its sclerosant effect on lymphatic endothelium (33, 34). However, therapeutic responses of bleomycin are always delayed for >3 months after initial treatment, and posttreatment accumulation of lymph may cause multiple sessions of treatment (35–37). Thus, for cLMs connecting to the lymphatic system, we think that inflow occlusion can inhibit lymph accumulation after sclerotherapy, so as to improve curative effect and reduce treatment frequency. Two hours after bleomycin sclerotherapy, we applied negative pressure for continuous drainage to promote cyst shrinkage and reduce posttreatment accumulation of the remaining lymph. Our technique provided rapid results, and 91.4% of cases did not need extra treatment postoperatively. Therefore, we believe that it could be an effective alternative for the management of macro or mixed cLM.

Our research has several limitations. Because ICG tissue penetration was limited to 10 mm for visualization, we cannot assure inflow occlusion in the deep tissue. Postoperative accumulation of lymph from deep inflows might be the cause of two cases with fair outcomes. In addition, further investigations with both larger sample sizes and longer follow-up period (1.5–2 years) would provide more concrete evidence for the effectiveness of this new technique.

In conclusion, this research introduces a novel technique, which combines inflow occlusion and bleomycin sclerotherapy based on intraoperative ICG lymphography. This procedure is performed with easy operation, satisfactory curative effect, and favorable treatment frequency. We therefore believe that the findings of this study could offer an effective and safe alternative for the management of macro or mixed cLM.

## Data Availability Statement

The original contributions presented in the study are included in the article/supplementary material, further inquiries can be directed to the corresponding author/s.

## Ethics Statement

The studies involving human participants were reviewed and approved by Ethics Committee of the Children's Hospital of Nanjing Medical University. Written informed consent to participate in this study was provided by the participants' legal guardian/next of kin. Written informed consent was obtained from the minor(s)' legal guardian/next of kin for the publication of any potentially identifiable images or data included in this article.

## Author Contributions

WS and JC performed the surgery and conducted the data analyses. YJ performed postoperative follow-up, analyzed the data, wrote sections of the article, and edited the figures. TH wrote a draft of the article and edited the figures. All authors contributed to the article and approved the submitted version.

## Funding

This study was supported by the Nanjing Medical Science and Technology Development Foundation (grant no. YKK20134).

## Conflict of Interest

The authors declare that the research was conducted in the absence of any commercial or financial relationships that could be construed as a potential conflict of interest.

## Publisher's Note

All claims expressed in this article are solely those of the authors and do not necessarily represent those of their affiliated organizations, or those of the publisher, the editors and the reviewers. Any product that may be evaluated in this article, or claim that may be made by its manufacturer, is not guaranteed or endorsed by the publisher.
